# A recurrent headache circumscribed in a coronal line-shaped area around the head: a coronal linear headache

**DOI:** 10.1186/s40064-016-1786-4

**Published:** 2016-03-10

**Authors:** Lei Wang, Jie-Feng Pan, Yun-Yun Lu, Liang-Hui Hu, Ya-Nan Lu, Qing-Qing Pan, Yu Wang

**Affiliations:** Department of Neurology, Epilepsy and Headache Group, the First Affiliated Hospital of Anhui Medical University, Jixi Road 218, Hefei, 230022 China; Department of Cardiology, The Second Division Korla Hospital of Xinjiang Production and Construction Corps, Jiaotong Road, Korla, 841000 Xinjiang China; Department of Neurology, The Second Division Korla Hospital of Xinjiang Production and Construction Corps, Jiaotong Road, Korla, 841000 Xinjiang China

**Keywords:** Linear headache, Coronal linear headache, Epicrania fugax, Nummular headache, Auriculotemporal neuralgia, Venous sinus, Migraine without aura

## Abstract

**Background:**

Linear headache (LH) has recently been described as a paroxysmal or continuous head pain restricted in a linear trajectory of 5–10 mm in width, linking one endpoint in occipital or occipitocervical region with another endpoint in ipsilateral nasion or forehead region. The sagittal line-shaped pain area of LH is close and parallel to a sagittal venous sinus, the superior sagittal sinus (SSS). For some patients, the LH had some features resembling the pattern of migraine without aura.

**Case description:**

A 45 year-old woman complained with a distinct headache for more than half years. The pain trajectory of the headache is confined to a coronal line-shaped area of 5–10 mm in width linking the two points in the bilateral temporal regions with the occipital protuberance. This coronal line-shaped pain area is close and parallel to a coronal cambered venous sinus complex, the combination of the confluences of sinus and the bilateral cavernous sinus (CS), superior petrosal sinus (SPS) linking the CS with transverse sinus (TS) and TS into which the SPS feeds. The patient had a past history of migraine without aura for 10 years and her son had a benign paroxysmal vertigo (BPV) for 12 years. Both of her coronal line-shaped headache and her son’s vertigo had well response to sodium valproate.

**Discussion and evaluation:**

Its clinical characteristics were distinctly different from those of other two headache entities defined with topographical criteria, nummular headache and epicrania fugax, and different from other existing headache entities except for migraine without aura.

**Conclusion:**

The distinct coronal line-shaped headache is suggestive of a variant of LH, a coronal LH, and probably belongs to a subtype of migraine without aura as proposed for LH. This coronal LH reinforces the proposal of LH as a new headache syndrome or a subtype of a previously known headache syndrome, probably of migraine.

## Background

Head pain of intracranial or extracranial, such as cranial neuralgia, cervicogenic headache (CEH), migraine and cluster headache (CH), may be confined within a restricted area. Such as, migraineurs may localize their pain to a particular area of occipital, parietal, frontal, and orbital region (Murtaza et al. 2009), nummular headache (NH) patients localize their pain to a restricted coin-shaped area on the head (Pareja et al. 2002). A headache area shaped in a linear trajectory but not confined to the territory of one particular nerve has been first reported in patients with epicrania fugax (EF) by Pareja et al. in (2008). EF is characterized by brief painful paroxysms starting in a particular area of the head, and rapidly radiating forward or backward along a linear trajectory innervated by different nerves, and its underlying pathophysiology is associated with peripheral origin (Pareja et al. 2008, 2012a; Guerrero et al. 2010; Cuadrado et al. 2013; Jin and Wang 2013). Wang et al. reported another head pain, linear headache (LH), restricted in a linear trajectory similar to that of EF, but its features significantly different from EF, are suggestive of central origin (Wang et al. 2014a). LH may represent a new headache syndrome or a variant of migraine as proposed based on its clinical features. LH patients present with a paroxysmal or chronic head pain restricted in a linear trajectory linking the occipital or occipitocervical region with the ipsilateral nasion or forehead region (Wang et al. 2014a, b). These two types of head pain both have a sagittal linear pain trajectory, here we report a distinct patient presenting with a recurrent head pain restricted in a coronal linear trajectory and had couple of features similar to that of LH. Thus, we may herein report another type of LH, reinforcing the proposal of LH as a new headache syndrome.

## Case description

A 45 year-old woman came to our department complaining of an episodic headache for more than half year and worsened for 2 weeks. A typical headache attack would start with a pulsating pain in the two pterions of the bilateral temporal regions. The bilateral pain would synchronously spread backward in a coronal way and converged at a midline point of the occipital area, the occipital protuberance, within ten minutes. After converging of the bilateral pain, the head pain would become fixed and restricted within this pain spreading trajectory, i.e. a line-shaped area of 5–10 mm in width linking the two pterions of the bilateral temporal regions and the occipital point. The pain, pulsating in characteristic, reached its peak in severity within 10–20 min and was described as moderate to severe, and the patient denied this pain epicranial but complain it intracranial. The headache attacking was often triggered or precipitated by anxiety but not related with menstruation. Dizziness, head heaviness, photophobia and phonophobia but not nausea or vomiting usually accompanied the headache attacking which would last for half day or remitted over a sleep. The headache usually attacked 3–4 times a month in the past half year, but attacked almost every day with occasional 1 day remission in recent 2 weeks. In recent 2 weeks, a typical coronal line-shaped head pain started with left side tinnitus which usually lasted for 10 min, otherwise the head pain and the left side tinnitus occured simultaneously, and the headache became more severe (7–8 in scale of 10) compared to those of the past half year (4–6 in scale of 10). Analgesics, a compound consisting of aminopyrine, phenacetin, caffeine and phenobarbital, in combination with flunarizine (5 mg once), may partially relieve the coronal line-shaped head pain in 5 min, but trial of the analgesics alone failed to relieve the pain. She had a history of migraine without aura attacks in a frequency of once per 2–3 months for more than 10 years while she was 20–30 year old, beginning after giving birth of her son and remitting 15 years ago. The migraine headache attacking was prone to be triggered by anxiety or dysphoria, and usually accompanied with dizziness and head heaviness, but with no nausea or vomiting. Partial relief of the headache could be achieved after taking analgesics, a compound consisting of aminopyrine, phenacetin, caffeine and phenobarbital, but a complete elimination of the headache achieved over a sleep.

There was no family history of migraine or other recurrent headaches, except for her son. Her son of 25 year old age, the first and the only one child, had two episodes of abrupt most severe (10 in scale of 10) lancinating pain over the whole head 4 and 2 years ago respectively. The episodes lasted for about 30 min and remitted spontaneously. According to the International Classification of Headache Disorders (ICHD-III, beta version) (IHS 2013), the headache was retrospectively diagnosed as thunderclap headache (TH). On the other hand, her son had recurrent severe vertigo attacks, a feeling of spinning around of the background, for 12 years in a frequency of 1–2 attacks per week, beginning at his age of 13 year old. Each episode of vertigo attacking would be preceded or accompanied with 1–2 min of amaurosis and the duration of the vertigo attacking would be 5–10 min. The vertigo attacking was usually accompanied with photophobia but with no phonophobia. No nausea or vomiting was noticed during the vertigo attacking and no headache attack found around the vertigo attacking. And he had a feeling of head heaviness and could not walk steadily while the vertigo was attacking. According to the International Classification of Headache Disorders (ICHD-III, beta version) ((IHS). 2013), the diagnosis of the episodic vertigo attacks could be grouped under “episodic syndromes that may be associated with migraine” in ICHD-III, beta version.

A complete physical and neurological examination was performed to exclude rhinitis, otitis media or cranial neuritis revealing normal. Inspection, palpation and sensory examination of the pain area, as well as the areas innervated by the lesser occipital nerve (LON), greater occipital nerves (GON) and auriculotemporal nerve (ATN) were all normal. Routine blood work-up with erythrocyte sedimentation rate (ESR), C-reactive protein (CRP), blood electrolyte and brain magnetic resonance imaging (MRI) and magnetic resonance venography (MRV) were all normal either.

The patient accepted prophylactic treatment with sodium valproate (500 mg twice a day) and her coronal line-shaped headache was dramatically alleviated leaving with only 1–2 times of mild headache attacks per month during the next three-month follow-up. Her son also accepted treatment with sodium valproate (500 mg twice a day) and his vertigo did not recur during the next 3 months follow-up.

## Discussion and evaluation

Here we report a patient presenting with a previously undescribed headache, a paroxysmal head pain restricted in a coronal linear trajectory, 5–10 mm in width, linking the two points in the bilateral temporal regions and the occipital protuberance. Apart from the line-shaped pain area similar to that of previously described LH and EF, all other features are obviously different from those of EF but apparently similar to those of LH and migraine. The clinical features of this new condition are suggestive of a new variant of LH and probably a distinct type of migraine without aura.

The current coronal line-shaped head pain is mainly defined with topographical criteria as did the EF, NH and LH. EF, a recently described novel headache syndrome, characterized by brief pain paroxysms starting in a particular area of the posterior scalp, and rapidly radiating forwards along a linear trajectory to reach the ipsilateral forehead, eye, or nose in a few seconds (Pareja et al. 2008, 2012a; Guerrero et al. 2010; Porta-Etessam et al. 2010; Mulero et al., 2011; Cuadrado et al. 2013; Jin and Wang 2013), has now been incorporated in the appendix of the third edition of the International Classification of Headache Disorders (ICHD-III beta) (IHS 2013; Belvis et al. 2015). A typical pain trajectory of EF is different from that of the currently reported coronal line-shaped head pain, whereas, recent reports have shown that the pain trajectory of EF may distribute in different area (de la Cruz et al. 2015), even in multiple directions (Cuadrado et al. 2015). Furthermore, Pareja and Bandres reported an interictal persistent line-shaped head pain in EF (Pareja and Bandres 2015). Thus, apparently, it should be considered that the current coronal line-shaped head pain may essentially be the interictal pain of EF. But the accompaniments of dizziness, head heaviness, photophobia and phonophobia, triggering factor of anxiety and response to flunarizine and sodium valproate make it obviously different from EF. In fact, this current coronal line-shaped head pain is much more consistent with the previously reported LH with the only difference in distributional direction, and the differences between LH and the interictal pain of EF had been listed in detail (Wang et al. 2015). Thus, we may conclude that the coronal line-shaped head pain is different from EF. Nummular headache (NH), another head pain defined with topographical criteria, was described first by Pareja et al. in 2002 (Pareja et al. 2002). NH is a continuous or intermittent pain which is commonly oppressive head pain circumscribed within a rounded or elliptical area, typically 1–6 cm in diameter (Pareja et al. 2002; IHS 2013). But, NH may present atypical features resembling a migraine pattern such as paroxymal episodes, accompaniments of nausea, photophobia and phonophobia (Dai et al. 2013), triggering or aggravating factor of physical exercise (Mulero et al. 2013; Baron et al. 2015), or relation to menstruation (Robbins and Grosberg 2010). Our patient also showed pain features resembling a migraine pattern of episodic pain accompanied with dizziness, head heaviness, photophobia and phonophobia, triggered by anxiety or dysphoria. Given the coronal line-shaped head pain be a special type of NH, the line-shaped pain area may be explained as an extension of a rounded or elliptical pain area of NH. And this is seemly supported by reports showing that NH pain sometimes affect multiple rounded areas on the head simultaneously (Cuadrado et al. 2009; Porta-Etessam et al. 2010; Guerrero et al. 2011; Rodriguez et al. 2015). Thus, series of NH pain areas may form a line-shaped pain area, otherwise, the small NH rounded pain area (1 cm or shorter in diameter) may expand in one direction forming a line-shaped area (5–10 mm in width) as described in the current coronal line-shaped head pain, or expand in all directions forming a large rounded area (6–8 cm in diameter) as reported in NH (Man et al. 2012; IHS 2013). Whereas, it is hard to explain how the pain affected a linear trajectory parallel to venous sinus of the transverse sinus (TS) in the current patient and the previously reported line-shaped head pain, the LH, is also parallel to venous sinus of the superior sagittal sinus (SSS) (Wang et al. 2014a, b), while the pain area can be localized in any part of the head in NH patients (Pareja et al. 2012b). Thus, we may conclude that the coronal line-shaped head pain is different from NH.

Auriculotemporal neuralgia (ATNa) is characterized by paroxysmal, moderate to severe pain on the preauricular area, often involving the ipsilateral temple and retro-orbital region (Speciali and Goncalves 2005). But the pain area of ATNa has never been reported to be line-shaped though its pain area could cover the coronal line-shaped head pain area given the bilateral ATNa could attack concurrently. In fact, ATNa is strictly unilateral pain (Stuginski-Barbosa et al. 2012) and the pain character is mainly lancinating and had never been reported accompanied with dizziness, head heaviness, photophobia and phonophobia as described in the current patient. Thus, the current coronal line-shaped head pain is significantly different from ATNa.

Except for the direction of the pain trajectory, all other features of the head pain in this woman are consistent with those of the previously reported LH in some patients, including the line-shaped pain area innervated by different nerves and the migrainous features of pulsating pain character, triggering or facilitating factor of anxiety, accompaniments of dizziness, head heaviness, and photophobia and phonophobia (Wang et al. 2014a). Thus this coronal line-shaped headache is suggestive of coronal LH. On the other hand, this patient had a past history of migraine headache attacking which was prone to be triggered by anxiety or dysphoria, and usually accompanied with dizziness and head heaviness. Further, her son had recurrent severe vertigo attacks for 12 years in a frequency of 1–2 attacks per week, beginning at his age of 13 year old. Each episode of vertigo attacking would be preceded or accompanied with 1–2 min of amaurosis and the duration of the vertigo attacking would be 5–10 min. And the recurrence of vertigo attacking was ceased by sodium valproate as reported in literature (Celiker et al. 2007; Cha 2010). This presentation can be diagnosed as benign paroxysmal vertigo (BPV) which is now grouped under the “episodic syndromes that may be associated with migraine” in ICHD-III beta (IHS 2013; Gelfand 2015), as he had ever accepted cranial CT examination excluding any occupying disease. Basing on these, we may conclude that this coronal LH might be associated with migraine.

It is unknown of the pathogenesis of the coronal LH. In previous report of LH case series, it is proposed that a line-shaped area of meningeal nociceptors is prone to be activated by cortical spreading depression (CSD), as the line-shaped pain area of LH is close and parallel to the superior sagittal sinus (SSS) and the immuno-vascular interactions causes a sensitization of the meningeal nociceptor (Levy 2012) parallel to the SSS (Wang et al. 2014a). An exciting finding in the present patient is the coronal LH pain area is also close and parallel to a coronal cambered venous sinus complex, the combination of the confluences of sinus (CFS) and the bilateral cavernous sinus (CS), superior petrosal sinus (SPS) linking the CS with transverse sinus (TS) and TS into which the SPS feeds. And the intracranial CS of bilateral sides are correspondent to the bilateral temporal areas where the coronal LH pain stemmed and the CFS correspondent to the converging area of pain spreaded from temporal areas of both sides. Thus, similar to that of LH, an immuno-vascular interaction may cause a sensitization of the meningeal nociceptor parallel to the cambered venous sinus complex and thus prone to be activated by CSD in our coronal LH patient, resulting in a coronal line-shaped cephalalgia through migraine pain pathway. This hypothesis is seemly supported by its positive response to sodium valproate which had been shown able to suppress CSD (Ayata et al. 2006), her past history of migraine and her son’s BPV associated with migraine.

## Conclusions

We encountered a patient with distinct headache presentations suggestive of a variant of LH, a coronal LH, and this coronal LH probably belongs to a subtype of migraine as proposed for LH. This headache description reinforces the proposal of LH as a new headache syndrome or a new variant of a previously known headache syndrome, probably of migraine.

## Patient consent

Signed consent is available from the patient for this report publication.

## Abbreviations

CEH: cervicogenic headache
CH: cluster headache
EF: epicrania fugax
NH: nummular headache
LH: linear headache
ICHD-III (beta version): 3rd edition (beta version) of the International Classification of Headache Disorders
TH: thunderclap headache
LON: lesser occipital nerve
GON: greater occipital nerve
ATN: auriculotemporal nerve
ESR: erythrocyte sedimentation rate
CRP: C-reactive protein
MRI: magnetic resonance imaging
ATNa: auriculotemporal neuralgia
BPV: benign paroxysmal vertigo
CSD: cortical spreading depression
SSS: superior sagittal sinus
TS: transverse sinus
CFS: confluences of sinus
CS: cavernous sinus
SPS: superior petrosal sinus
